# Teenage drinking, alcohol availability and pricing: a cross-sectional study of risk and protective factors for alcohol-related harms in school children

**DOI:** 10.1186/1471-2458-9-380

**Published:** 2009-10-09

**Authors:** Mark A Bellis, Penelope A Phillips-Howard, Karen Hughes, Sara Hughes, Penny A Cook, Michela Morleo, Kerin Hannon, Linda Smallthwaite, Lisa Jones

**Affiliations:** 1 Centre for Public Health, Faculty of Applied Health and Social Science, Liverpool John Moores University, Fifth Floor Kingsway House, Hatton Garden, Liverpool L3 2AJ, UK; 2 Halton Consumer Protection Service, Rutland House, Halton Lea, Runcorn WA7 2GW, UK

## Abstract

**Background:**

There is a lack of empirical analyses examining how alcohol consumption patterns in children relate to harms. Such intelligence is required to inform parents, children and policy relating to the provision and use of alcohol during childhood. Here, we examine drinking habits and associated harms in 15-16 year olds and explore how this can inform public health advice on child drinking.

**Methods:**

An opportunistic survey of 15-16 year olds (n = 9,833) in North West England was undertaken to determine alcohol consumption patterns, drink types consumed, drinking locations, methods of access and harms encountered. Cost per unit of alcohol was estimated based on a second survey of 29 retail outlets. Associations between demographics, drinking behaviours, alcohol pricing and negative outcomes (public drinking, forgetting things after drinking, violence when drunk and alcohol-related regretted sex) were examined.

**Results:**

Proportions of drinkers having experienced violence when drunk (28.8%), alcohol-related regretted sex (12.5%) and forgetting things (45.3%), or reporting drinking in public places (35.8%), increased with drinking frequency, binge frequency and units consumed per week. At similar levels of consumption, experiencing any negative alcohol-related outcome was lower in those whose parents provided alcohol. Drunken violence was disproportionately associated with being male and greater deprivation while regretted sex and forgetting things after drinking were associated with being female. Independent of drinking behaviours, consuming cheaper alcohol was related to experiencing violence when drunk, forgetting things after drinking and drinking in public places.

**Conclusion:**

There is no safe level of alcohol consumption for 15-16 year olds. However, while abstinence removes risk of harms from personal alcohol consumption, its promotion may also push children into accessing drink outside family environments and contribute to higher risks of harm. Strategies to reduce alcohol-related harms in children should ensure bingeing is avoided entirely, address the excessively low cost of many alcohol products, and tackle the ease with which it can be accessed, especially outside of supervised environments.

## Background

In recent decades alcohol has emerged as one of the major international threats to public health [1], and is now the third largest risk factor for disability and death in Europe [2]. Alcohol alone is thought to be responsible for 4.0% of the global burden of disease [3] with Europe having higher levels of consumption per person than any other global region [4,5]. As a result Europe suffers 195,000 deaths relating to alcohol each year [5], amounting to 6.1% of all deaths and 12.3% of all years of life lost [6]. Despite much of the chronic burden of alcohol-related disease falling on adults [7], the foundations of such damage are often established in childhood. Early alcohol initiation (e.g. before age 15) [8,9] and drinking in larger quantities in childhood and adolescence [10,11] are associated with a wide range of negative outcomes including initiation of drug use, suicide ideation, delinquency, violence, injury, depression and school absenteeism. Such drinking also increases the risks of developing chronic health and other problems (e.g. alcohol dependency, illicit drug use, liver disease) in later life [12-14]. Those initiating alcohol use before the age of 13 are particularly vulnerable to adverse health outcomes [8,9].

Misuse of alcohol by children is an international problem. Pan-European studies report that between 35% (Isle of Man) and 2% (Armenia) of 15-16 year olds have been drunk at least once in the past 30 days [15]. Further, a substantial proportion have binged (five or more drinks in one session) three or more times over the same period (ranging from 34% in the Isle of Man to 8% in Iceland and Romania) [15]. By both survey measures, the UK shows high levels of alcohol misuse by youths (33% and 27% respectively). Moreover, recent trends suggest such problems have increased in the UK with the average weekly quantity of alcohol consumed by 11-15 years old drinkers having doubled (1990-2008) [16] and the number of children under 16 admitted to hospital (with diagnoses specific to alcohol) increasing by 29% (1995/96-2005/06) [17]. Such increases in alcohol-related ill health in children are not restricted to the UK (e.g. Germany [18], Australia [19]).

Despite considerable acute and chronic health and social consequences relating to child alcohol consumption, evidence based guidance on whether children should drink alcohol at all, and how to moderate potential harm, is still being sought [20]. In particular, the effects of moderate or occasional consumption are unclear. Thus, while drinking at early ages (under 15 years) is linked to experiencing a range of health and social problems, the effects of alcohol use at age 15 can depend on amounts consumed, frequency of consumption, types of alcohol consumed and the context in which consumption takes place [21,22]. Alcohol illicitly obtained by children is associated with misuse [23]. However, alcohol provided by parents has been associated with reduced involvement in binge drinking and drinking in public places [23,24] compared with other means of access, and strict alcohol-specific parenting rules have been associated with reduced consumption [25-27]. However, in those aged 12, easy access to alcohol from parents is associated with increased alcohol abuse [28] and parental provision for parties has been linked to increased drinking [24]. With no clear understanding of the relationships between drinking behaviours, environments where alcohol is accessed and consumed, and resultant harms, more research is urgently needed to examine how such factors interact and to inform appropriate interventions.

In this paper we examine the drinking behaviours of alcohol-consuming 15-16 year olds and their relationships with a range of adverse alcohol-related outcomes. Thus, based on previous associations between alcohol consumption and violence [29] we examine experience of violence when drunk and how it relates to current drinking behaviours. With greater alcohol consumption at early ages also being associated with sexual risk-taking [30,31], we explore relationships between drinking behaviours and having experienced regretted sex following alcohol consumption. As a proxy measure of potential damage to mental health we analyse associations between drinking patterns and reported tendency to forget things after drinking [32]. Finally, to measure effects on others through public nuisance and potentially anti-social behaviour, we examine which drinking patterns are associated with consumption in public places (here; outside in streets, around shops and in parks). Together, analyses are also used to examine potential thresholds for safer drinking and explore factors that may moderate relationships between consumption and immediate harms. Finally, by examining the types of alcohol products individuals consume we also explore which drinking behaviours are associated with consumption of particular products.

## Methods

### Questionnaire design

The North West Region (population, 6,840,000) [33] suffers some of the highest levels of alcohol-related harm in England [34]. Consequently, an anonymous school based survey was undertaken across this Region, led by Trading Standards North West, to examine the drinking behaviours of its residents. Building on a survey tool developed and utilised in 2005 [23], the questionnaire consisted of closed, self-completed questions including: demographics (age, sex and postcode of residence); usual frequency of alcohol consumption and bingeing (here, drinking five or more drinks in one session [15]); and how individuals accessed alcohol and types of alcohol products consumed in a typical week (e.g. cans of beer, bottles of wine). For alcohol types consumed, respondents were provided with short descriptions and small pictures of typical products to help with identification. The types of alcohol products listed were based on those in established national surveys [35]. Individuals were also asked to identify if they drank alcohol in public places and these were described to respondents as outside in streets, parks or shops. The questionnaire asked respondents to identify (by tick box) if they had ever been violent or in a fight whilst drunk; whether they had regretted having had sex with someone after drinking; and whether they tended to forget things when they had been drinking alcohol. For regretted sex after drinking, the questionnaire did not distinguish between those who were sexually active but had never had regretted sex after drinking and those who were sexually inactive. Both were considered positive outcomes compared with having had regretted sex related to alcohol consumption. To analyse the question 'I tend to forget things when I have been drinking alcohol', a four point ordinal Likert Scale (agree strongly, agree, disagree, disagree strongly) was dichotomised into those that agreed that they tended to forget things after drinking and those that did not. Income was calculated from three questions identifying monies obtained from parents, work and other sources. For access to alcohol, variables measured were: personal purchase from on- and off-licence settings; access through parents, friends and family; and proxy purchasing through other adults. Access through parents distinguished between deliberate provision of alcohol by parents and alcohol covertly taken by youths.

### Questionnaire delivery

The questionnaire was made available to secondary schools across the North West for whom participation was voluntary. Students were informed that participation was voluntary and anonymous and data were collected solely for the purpose of aggregated analyses. All aspects of the research methodology complied fully with the Helsinki Declaration. The survey (run every two years) was established by Local Authority Trading Standards in the North West and was scrutinised and approved by the Trading Standards North West Executive committee and supported by the cross-departmental Alcohol Forum at Government Office North West. Formal ethical approval was not requested in 2007 as this survey is an ongoing biennial process established by Trading Standards in 2005 (in agreement with public sector partners) as an audit of their role in preventing alcohol sales to minors. Sampling was not intended to be representative of all students across the North West but was designed to encompass a wide range of community types. School staff delivered questionnaires to students within normal school hours in years 10 and 11 (including individuals aged 14 to 17 years) [23] with classrooms being surveyed on an opportunistic basis. Previous North West surveys of youth alcohol consumption provided appropriate sample sizes (target 10,000 respondents [23]) and sampling targeted an age range typically associated with the early stages of routine alcohol use [15,16]. Sampling was completed after a total of 140 schools across 19 local authorities in the North West had participated providing 11,724 questionnaires (between January and March 2007). For the purposes of analyses undertaken here, the sample was then restricted to those aged 15 or 16 (n = 9,833). Response rates were not recorded in each class as the sample was not intended to be representative but was opportunistic (for both students and classroom participation), with analyses focusing on relationships between variables recorded by individual participants. To study drinking behaviour the sample was further limited only to those who identified that they drank alcohol (n = 8,263; 84%). Individuals who did not drink were only excluded at this stage (cf. at the point of questionnaire distribution) so that those who drank would not have to reveal this in class.

### Respondent deprivation classification

Using an ecological methodology, all individuals were allocated to a quintile of deprivation across the North West. Index of Multiple Deprivation (IMD) [36] has been calculated for all Lower Super Output Areas (LSOAs) in England. LSOAs are geographical areas with an average population size of approximately 1,500 individuals and are the smallest areas for which an index of deprivation have been calculated across England [37]. Individuals were allocated directly to a LSOA by full postcode when provided (n = 4,158) with postcodes being mapped directly to LSOA geographical boundaries. Those pupils providing partial postcodes (which spanned more than one LSOA) were allocated to a LSOA on the basis of which LSOA contained the majority of postcodes possible within the partial postcode provided (n = 1,744). A further 2,063 individuals provided no postcode and therefore school postcode was used as a proxy deprivation geography [23]; a method which has been successfully used elsewhere [38]. Furthermore, in our sample for those respondents providing a postcode of residence, deprivation scores by postcode of residence correlated with deprivation scores by postcode of school (P < 0.001). However, LSOA (and therefore deprivation) was calculated from individuals' specific postcodes of residence rather than the more general school postcodes when both were available. Once LSOA was established for each individual, they were categorised into deprivation quintiles according to where their LSOA fell in the list of all LSOAs in the North West ranked by deprivation. Questionnaires providing insufficient data for any method of geographical classification (n = 298) were excluded from geographic analyses.

### Retail costs of alcohol types

The retail price of each alcohol product type described on the questionnaire was collected from 29 off-licence venues. Sampling included supermarkets, off-licences and other licensed shops within the residential boundaries of the school sample. Although not all underage drinkers may select the cheapest alcohol (e.g. product status may also affect choice), based on other studies we hypothesised that economic pressures may result in the heaviest drinkers being the most price sensitive in their drink selection [39]. Therefore, in each outlet mystery shoppers were asked to identify the cheapest (cost per unit of alcohol) example of each product type and record the volume, price and alcohol content. Items were priced based on individual or multi-pack costs (e.g. bottle of wine or four-pack of beers). Price reductions for larger bulk buys (e.g. 40 cans of beer or six bottles of wine) were excluded. In total, seven different product types were sampled (alcopops, regular bottles/cans of beer, regular bottles/cans of cider, bottles of wine, bottles of spirits and large multi-litre value bottles of cider and of beer). Cost per unit of alcohol for each product was calculated from its volume, alcohol concentration and retail value. For each product type, costs per unit of alcohol were then averaged across all retailers. However, large multi-litre bottles of beer were excluded from product analyses as few respondents reported drinking them and most retail outlets did not sell them.

### Calculating weekly alcohol consumption

To estimate weekly consumption, the alcohol products listed on the questionnaire were converted into standard units (1 unit = 8 grams or 10 ml of pure alcohol) consumed using: an alcopop (bottle) = 1.5; bottle or can of beer = 2; bottle or can of cider = 2; glass of wine (or quarter of a bottle) = 2.5; shot of spirits = 1; large value cider (2 litres) = 10.5 and large value beer (2 litres) = 10.5 units (based on updated units per drink methodologies [35]). An open question allowed individuals to list other less commonly consumed products (e.g. a liqueur). These were also converted into units based on alcohol contents typical of each product. As questions only addressed numbers consumed during a typical week, those drinking less than once a week were excluded from analyses relating to units per week consumed. The lack of consumption data on those drinking less than weekly means this variable was excluded from logistic regression models. All data were entered into SPSS v14 by Ci Research and sent for cleaning and analysis at Liverpool John Moores University. Analyses utilised Chi square, Spearman's correlation, ANOVA and backward conditional Logistic Regression techniques.

All individuals answered questions on age and gender as well as those on sources of alcohol consumed (e.g. buy own, parents provide, from adults outside shop). For other variables utilised, completeness of data was: weekly income 88.1%; binge frequency 98.8% and drinking frequency 99.9%. Units consumed per week were only calculable for those drinking at least weekly and for such individuals estimates were possible for 81.2% of respondents. Data completeness for negative outcome dependent variables was: drink outside 100%; alcohol-related violence 95.7%; alcohol-related regretted sex 90.8% and; tend to forget things after drinking 96.6%.

## Results

Regretted sex after drinking (12.5%), having been involved in violence when drunk (28.8%), consuming alcohol in public places (e.g. streets, parks and shops; 35.8%) and forgetting things after drinking (45.3%) had all been experienced by relatively large proportions of respondents. Violence when drunk and alcohol-related regretted sex both increased with age (Table 1). While violence when drunk and drinking in public places were more common amongst boys, alcohol-related regretted sex and forgetting things after drinking were more commonly reported by girls. Proportions who drank in public places, experienced violence when drunk and regretted sex after drinking all increased with deprivation. However, forgetting things after drinking showed no such relationship. Having a higher weekly income was positively associated with all adverse outcomes as were respondents buying their own alcohol or asking adults outside retail venues to buy it for them (i.e. proxy purchasing; Table 1). Importantly, accessing alcohol through parents was associated with significantly lower levels of having experienced all negative outcomes (Table 1).

**Table 1:** Relationships between demographics, sources of alcohol and percentage of children aged 15 to 16 years having experienced negative alcohol-related outcomes

			**n**	**Drink in public places (streets, parks, shops)**	**Violence** **when drunk**	**Alcohol-related regretted sex**	**Tend to forget things after drinking**
Age in Years		15	4026	36.0	26.9	11.0	45.2
		16	4237	35.6	30.6	14.0	45.4
		P		0.671	<0.001	<0.001	0.883

Sex		Female	4303	34.0	25.8	13.3	50.0
		Male	3960	37.8	32.1	11.7	40.1
		P		<0.001	<0.001	<0.05	<0.001

Deprivation Quintile		(Wealthiest) 1	1275	32.6	22.4	10.2	44.4
		2	1687	32.1	26.4	11.1	46.1
		3	1439	38.6	28.2	14.0	44.1
		4	1597	37.0	31.4	12.7	46.0
		(Poorest) 5	1954	38.4	32.7	13.9	44.9
		P		<0.001	<0.001	<0.01	0.728

Weekly Income		< = £10	2584	34.4	22.7	9.4	40.7
		>£10-20	2064	37.3	29.7	11.4	48.5
		>£20-30	1035	34.3	32.7	13.6	47.5
		>£30	1593	41.6	38.3	20.1	48.4
		P		<0.001	<0.001	<0.001	<0.001

Source	Buy my own	No	5923	32.15	22.41	8.86	42.61
		Yes	2340	45.00	44.82	21.39	51.98
		P		<0.001	<0.001	<0.001	<0.001
	
	Parents	No	4182	47.0	37.1	15.3	51.4
	provide	Yes	4081	24.3	20.3	9.6	39.1
		P		<0.001	<0.001	<0.001	<0.001
	
	Get adults	No	7060	27.9	24.7	11.1	42.8
	outside shop	Yes	1203	82.2	52.4	20.8	59.7
	to buy it	P		<0.001	<0.001	<0.001	<0.001

Negative drinking outcomes were also strongly associated with the types of alcohol products respondents consumed in a typical week. Thus, while only 34.1% of those drinking wine drank in public places, this increased to over 70% amongst those who drank large value cider bottles (Table 2). In fact, higher proportions of large value cider and spirits drinkers had suffered alcohol-related regretted sex, violence when drunk and forgetting things after drinking compared with drinkers of other products (e.g. alcopops; Table 2). Correlation was used to examine whether consumption of lower priced drinks was related to greater percentages of consumers experiencing negative alcohol-related outcomes. Results suggest a strong relationship between consumption of cheaper alcohol products and increased proportions of respondents reporting violence when drunk, alcohol-related regretted sex and drinking in public places (Table 2).

**Table 2:** Relationships between types of alcohol products consumed, costs per unit of alcohol for each product type and percentage of children having experienced negative alcohol-related outcomes

	**% consuming**	**Drink outside (streets, parks, shops)**			**Violence when drunk**			**Alcohol-related regretted sex**			**Tend to forget things after drinking**			**Price per unit of alcohol (£)**			
**Drink product**	**drink type**	**%**	**OR**	**95% CIs**	**%**	**OR**	**95% CIs**	**%**	**OR**	**95% CIs**	**%**	**OR**	**95% CIs**	**n**	**Mean**	**95% CIs**	**Lowest**
Alcopops	50.72	40.15	1.02	0.92-1.14	30.40	0.90	0.80-1.00	14.58	1.15	0.99-1.34	49.90	1.21	1.09-1.34	24	0.70	0.61-0.78	0.33
Beer cans or bottles	56.35	44.53	1.57	1.41-1.75	37.74	1.97	1.75-2.22	15.61	1.45	1.23-1.70	47.91	1.03	0.93-1.14	26	0.37	0.34-0.41	0.28
Wine	26.62	34.10	0.72	0.63-0.81	31.17	0.98	0.86-1.11	16.01	1.28	1.08-1.52	48.22	1.04	0.92-1.16	25	0.37	0.34-0.40	0.23
Spirits	48.43	48.63	2.05	1.84-2.28	41.35	2.47	2.20-2.77	18.28	2.16	1.84-2.54	54.21	1.68	1.51-1.87	29	0.33	0.30-0.35	0.21
Cider cans or bottles	22.11	49.81	1.69	1.49-1.91	37.90	1.44	1.27-1.64	17.02	1.39	1.17-1.66	51.01	1.19	1.05-1.35	23	0.28	0.24-0.31	0.15
Large value cider bottles	12.71	71.56	4.62	3.91-5.47	50.48	2.53	2.16-2.96	24.02	2.27	1.87-2.75	60.79	1.85	1.58-2.16	22	0.17	0.16-0.19	0.11

P (correlation)^$^		<0.01			<0.01			<0.01			<0.05			<0.001^#^			

^$^P relates to correlation between percentage experiencing each negative alcohol-related outcome by drink type and mean price per unit of alcohol by drink type. Correlations use Spearman's _(one tailed) _tests to examine the hypothesis that consumption of lower priced drinks are related to greater percentages of consumers experiencing harms. Odds ratios (OR) and 95% confidence intervals (95% CIs) measure the relative increase in odds of having experienced each negative alcohol-related outcome associated with being a consumer of each drink product. As individuals often were consumers of more that one drink product the same individual can appear in the analysis of more than one drink product type. ^#^Differences between prices of each product utilises ANOVA.

Table 3 presents the relationship between three reported drinking measures (units per week, frequency of drinking, and of bingeing) and proportions reporting each negative outcome overall and separately for those who do and do not have alcohol provided by parents. Overall, all negative outcomes increased in frequency significantly as drinking frequency, bingeing frequency and units of alcohol consumed per week increased. However, provision of alcohol by parents was associated with lower levels of harm at the same drinking and bingeing frequency, and at the same weekly quantities of consumption. Thus, while 19.9% of individuals whose parents provide alcohol and who drink once a week had been involved in violence when drunk, this rises to 35.9% in those whose parents do not provide alcohol (Table 3). Similarly for those without parental provision of alcohol, 15.2% of those who drink up to five units of alcohol per week reported some alcohol-related regretted sex, while for those with parental provision rates are only 11.7% even at >10-20 units per week (Table 3). However, such protective effects were not sustained across all adverse outcomes at higher levels of consumption (especially at high levels of binge drinking).

**Table 3:** Percentage of 15-16 year olds having experienced negative alcohol-related outcomes, by drinking behaviour and parental alcohol provision

					**Percentages reporting negative outcomes related to alcohol**															
					
		**Sample Characteristics**			**Drink in public places (streets, parks, shops)**				**Violence when drunk**				**Alcohol-related** **regretted sex**				**Tend to forget things after drinking**			
		**Parents Provide**			**All**	**Parents Provide**			**All**	**Parents Provide**			**All**	**Parents Provide**			**All**	**Parents Provide**		
		**n**	**No**	**Yes**		**No**	**Yes**	**P^§^**		**No**	**Yes**	**P^§^**		**No**	**Yes**	**P^§^**		**No**	**Yes**	**P^§^**
Binge Frequency	Never	1007	36.4	63.6	11.2	24.0	3.9	***	7.1	13.0	3.7	***	3.8	6.9	2.0	***	21.6	32.4	15.6	***
	<1/month	2302	43.1	56.9	21.4	33.1	12.5	***	13.6	21.0	8.1	***	6.1	8.0	4.7	**	36.6	43.7	31.2	***
	1-3/month	1894	48.9	51.1	34.2	43.5	25.3	***	24.6	30.9	18.6	***	8.6	9.8	7.4	ns	47.4	51.5	43.7	***
	1/week	1533	60.9	39.1	48.9	55.0	39.5	***	40.0	42.9	35.4	**	15.4	16.6	13.5	ns	54.9	55.6	53.8	ns
	2/week	1173	62.4	37.6	64.5	69.3	56.7	***	59.8	63.6	53.6	***	28.4	29.0	27.4	ns	61.9	62.4	60.9	ns
	3+/week	254	65.0	35.0	63.4	61.8	66.3	ns	72.4	75.5	66.7	ns	39.1	39.5	38.3	ns	63.6	66.7	58.1	ns
	P		***		***	***	***		***	***	***		***	***	***		***	***	***	

Drinking Frequency	<1/month	1750	44.2	55.8	14.9	24.3	7.5	***	10.6	16.5	6.1	***	4.7	5.4	4.2	ns	31.6	39.6	25.5	***
	1-3/month	2097	46.8	53.2	27.2	37.8	17.9	***	17.9	24.3	12.3	***	7.1	8.5	5.9	*	40.8	46.1	36.3	***
	1/week	2041	53.7	46.4	40.7	52.1	27.5	***	28.4	35.9	19.9	***	11.0	13.5	8.2	***	47.5	52.1	42.2	***
	2/week	1791	56.9	43.1	54.3	63.5	42.2	***	48.4	55.8	38.8	***	21.7	24.3	18.3	**	57.5	61.0	53.0	***
	3+/week	575	53.0	47.0	55.8	62.3	48.2	***	61.7	69.9	52.1	***	30.2	36.3	23.0	***	56.1	63.1	48.5	***
	P		***		***	***	***		***	***	***		***	***	***		***	***	***	

Units per week^$^	< = 5	469	39.9	60.1	27.1	51.3	11.0	***	18.2	33.7	8.4	***	9.4	15.2	5.8	**	36.5	47.2	29.5	***
	>5-10	700	41.7	58.3	29.7	41.1	21.6	***	20.4	29.5	13.9	***	8.8	13.6	5.4	***	42.7	52.1	35.9	***
	>10-20	1106	51.9	48.1	45.6	54.9	35.5	***	35.1	40.9	28.8	***	13.2	14.5	11.7	ns	56.1	57.2	55.0	ns
	>20-30	604	59.8	40.2	60.1	67.3	49.4	***	55.3	57.8	51.5	ns	21.4	19.8	23.8	ns	57.7	58.0	57.2	ns
	>30	700	60.4	39.6	68.1	72.1	62.1	**	64.9	69.0	58.8	**	32.7	36.3	27.4	*	59.5	60.8	57.5	ns
	P		***		***	***	***		***	***	***		***	***	***		***	*	***	

P^§^compares those whose parents provide and do not provide any alcohol for proportions having experienced each negative risk behaviour within categories of units per week, drinking and binge drinking frequency. * P < 0.05, ** P < 0.01, *** P < 0.001. ^$^Units per week consumed could only be calculated for those reporting a drinking frequency of once per week or greater and for those individuals providing details of types of alcohol products consumed and quantities of each product consumed in a typical week.

Finally, logistic regression analysis was used to examine factors relating to having experienced negative alcohol outcomes while controlling for confounding relationships between sources of alcohol, types consumed, drinking patterns and individuals' demographics. Here, frequency of binge drinking remained strongly related to having experienced all negative outcomes (Table 4). However, compared with drinking less than once a month, drinking at greater frequency was only related to having been involved in violence when drunk and drinking in public places. Independent of drinking and binge frequency, typically consuming multi-litre value cider bottles was associated with increased risks of all negative outcomes. Equally, spirits consumption was related to increases in all risks except regretted sex and drinking standard bottles and cans of beer to all except forgetting things after drinking (Table 4). Importantly, wine consumption was associated with less public drinking and alcopops with less violence when drunk. Source of alcohol was also an important factor, with accessing alcohol through proxy purchasing increasing risks of all negative outcomes and parental provision being associated with reduced risks. Respondents' personal income was positively related to risks of having experienced alcohol-related regretted sex and violence (Table 4). However, deprivation was only associated with violence when drunk. Thus, those in the poorest quintile were at highest risks even after adjustments for drinking and binge frequency (Table 4). Increasing age was related to a small but significant decrease in proportions drinking in public places and finally, females were more likely to report regretted sex and especially forgetting things as negative outcomes of drinking, while males were more likely to report violence (Table 4).

**Table 4:** Logistic regression analysis examining negative outcomes from alcohol consumption by 15 and 16 year olds and their relationship with demographics, drinking behaviour and sources of alcohol

			**Drink in public places (streets, parks, shops)**				**Violence when drunk**				**Alcohol-related regretted sex**				**Tend to forget things after drinking**			
			
			**AOR**	**95% CIs**		**P**	**AOR**	**95% CIs**		**P**	**AOR**	**95% CIs**		**P**	**AOR**	**95% CIs**		**P**
			
		Sex (Male)	ns				1.18	1.03	1.35	<0.05	0.70	0.59	0.83	<0.001	0.64	0.58	0.71	<0.001
		Age (16 years)	0.89	0.79	1.00	<0.05	ns				ns				ns			

Deprivation	Wealthiest	1 (ref)	ns							<0.001	ns				ns			
quintile		2					1.21	0.98	1.49	0.069								
		3					1.23	1.00	1.52	0.052								
		4					1.49	1.21	1.83	<0.001								
	Poorest	5					1.47	1.20	1.79	<0.001								

Weekly		< = £10 (ref)	ns							<0.001				<0.001				<0.05
income		>£10-20					1.09	0.93	1.27	0.302	0.95	0.77	1.18	0.647	1.23	1.08	1.39	<0.01
		>£20-30					1.35	1.12	1.62	0.002	1.20	0.94	1.54	0.140	1.15	0.98	1.35	0.077
		>£30					1.35	1.14	1.59	<0.001	1.48	1.20	1.82	<0.001	1.07	0.93	1.23	0.340

Source^$^		Buy my own	ns				1.55	1.36	1.76	<0.001	1.83	1.55	2.15	<0.001	ns			
		Parents provide	0.51	0.45	0.57	<0.001	0.57	0.50	0.65	<0.001	0.75	0.64	0.88	<0.001	0.75	0.68	0.83	<0.001
		Get adults outside shop to buy it	7.79	6.51	9.32	<0.001	2.13	1.82	2.49	<0.001	1.48	1.22	1.80	<0.001	1.40	1.21	1.62	<0.001

Drink type		Alcopops	ns				0.82	0.72	0.94	<0.01	ns				ns			
consumed^$^		Beer Cans or Bottles	1.39	1.24	1.57	<0.001	1.24	1.08	1.43	<0.01	1.24	1.04	1.47	<0.05	ns			
		Cider Cans or Bottles	ns				ns				ns				ns			
		Wine	0.77	0.66	0.89	<0.001	ns				ns				ns			
		Spirits	1.44	1.28	1.63	<0.001	1.49	1.31	1.71	<0.001	ns				1.22	1.10	1.36	<0.001
		Large Value Cider Bottles	2.78	2.27	3.40	<0.001	1.29	1.07	1.56	<0.01	1.39	1.12	1.73	<0.01	1.31	1.10	1.57	<0.01

Drinking		Less than once a month (ref)				<0.001				<0.01	ns				ns			
frequency		1-3 times a month	1.39	1.11	1.75	<0.005	0.97	0.74	1.25	0.796								
		Once a week	1.71	1.34	2.17	<0.001	1.05	0.80	1.38	0.724								
		Twice a week	1.76	1.33	2.31	<0.001	1.35	1.00	1.81	0.050								
		3+ Times a week	1.65	1.14	2.38	<0.01	1.84	1.26	2.68	<0.01								

Binge		Never (ref)				<0.001				<0.001				<0.001				<0.001
frequency		Less than once a month	1.83	1.41	2.37	<0.001	1.85	1.36	2.52	<0.001	1.37	0.90	2.09	0.143	1.97	1.62	2.40	<0.001
		1-3 times a month	2.55	1.94	3.36	<0.001	3.05	2.19	4.23	<0.001	1.65	1.09	2.51	<0.05	2.80	2.29	3.43	<0.001
		Once a week	3.24	2.43	4.34	<0.001	4.47	3.19	6.28	<0.001	2.63	1.73	3.98	<0.001	3.62	2.93	4.48	<0.001
		Twice a week	5.46	3.93	7.57	<0.001	7.34	5.08	10.60	<0.001	5.27	3.48	7.96	<0.001	4.72	3.77	5.91	<0.001
		3+ Times a week	4.15	2.52	6.83	<0.001	7.32	4.35	12.34	<0.001	7.09	4.31	11.64	<0.001	4.49	3.16	6.38	<0.001

AOR = Adjusted Odds Ratio; 95% CIs = 95% Confidence Intervals; ns = not significant. ^$^Categories in these sections are separate binary variables (e.g. alcopop consumer yes/no, beer in cans or bottles consumer yes/no, buy my own yes/no, etc) and so are included in the model as separate variables. Reference categories thus consist of persons not reporting having consumed that drink, and not accessing alcohol from that source.

## Discussion

Consistent with studies in the USA [11,29], our results show that substantial proportions of even those that drink at relatively low frequencies (e.g. weekly) or never binge have experienced adverse effects. Thus, 10.6% of individuals who drink less than once a month have still experienced violence when drunk and nearly a third report forgetting things after drinking (Table 3). However, amongst children whose parents provide alcohol, violence when drunk and forgetfulness drop to 6.1% and 25.5% in such lower frequency drinkers. Previous studies suggest that both parental attitudes towards, and their supervision of youth drinking can affect young people's drinking behaviours [23-28]. However, results here suggest that similar drinking patterns are more likely to be related to adverse outcomes when alcohol is accessed outside of parental environments. Thus, as well as drinking frequency, parental provision also appears to have a mediating effect on risks associated with binge drinking and units consumed per week (Table 3). However, any protective effects are limited. Thus, 35.4% of those bingeing once a week, even with parental provision, have been involved in violence when drunk (Table 3) and amongst respondents reporting the highest frequency of binge drinking, protective effects of parental provision disappear (Table 3). However, as we were unable to differentiate types of parental provision (e.g. for unsupervised parties or consumption at family meals), here we cannot identify specifically how context relates to risks.

With 84.0% of 15 and 16 year olds surveyed already consuming alcohol we have analysed the data to quantify the relationship between increased consumption and changes in risk of adverse outcomes. After correcting for confounding factors, risks for drinking in public places increase as frequency of consumption increases. However, differences in risks of involvement in violence when drunk only approach significance when drinking frequencies exceed once a week (compared with drinking less than once a month). Our results identify that bingeing at any frequency (c.f. those that drink but never binge) is associated with significantly higher levels of violence when drunk, tendency to forget things after drinking and drinking in public places (Table 4). Alcohol-related regretted sex was also associated with bingeing but increased risks (compared with never bingeing) only escalated significantly at binge frequencies of one to three times a month or more.

Overall, results suggest any binge drinking by 15 and 16 year olds should be avoided. Such findings are supported by neurocognitive studies, which have found underage heavy episodic or binge drinking to be associated with brain damage as adolescent brains are more susceptible to neurochemical changes, neurodegeneration and long-lasting changes in functional activity [32,40]. However, a recent review of the evidence suggests that the precise risks that alcohol consumption represents to the adolescent brain are still unclear [41]. Our results, even after correcting for binge and drinking frequency, identify an independent association between tendency to forget things after drinking and being female (Table 4). Such damage may now be exacerbated by young females' consumption of alcohol in the UK approaching the same level as males [16].

While all adverse outcomes increased with weekly units consumed (Table 3) not all were significantly different between < = 5 and >5-10 units/week categories. Thus, proportions of respondents having experienced violence, regretted sex and drinking in public places did not differ significantly (P = 0.364; 0.734; 0.329 respectively) between < = 5 and >5-10 unit categories. However, forgetting things did show a significant increase (P < 0.05). At >10-20 units/week all negative outcomes were significantly higher than both < = 5 and >5-10 unit categories. Consequently, while teenage drinkers may experience similar behavioural risks while increasing consumption up to 10 units/week, effects on tendency to forget things appear to increase with consumption at all levels. However, our results suggest types of alcohol consumed may mitigate or aggravate alcohol-related harms. Consuming value multi-litre cider was strongly linked with increases in all risks, and consuming spirits with all except regretted sex (Table 4). Both value cider and spirits purchases often result in having large amounts of alcohol in a single bottle. Whilst our study did not examine how such products were consumed, a single bottle may encourage individuals to consume the contents more quickly or, where sharing occurs (e.g. passing around the bottle), rapidly consume greater quantities on their turn. Furthermore, drinking may finish only when the contents are exhausted. Importantly, both products were two of the cheapest ways of purchasing units of alcohol. Cider provided alcohol for as little as £0.11 per unit (Table 2) meaning that consuming five units (more than adult daily recommended levels in the UK) was comparable with the price of a can of a popular cola. By contrast alcopops provide a relatively expensive cost per unit of alcohol, having typically been sold in smaller volume containers. In our analyses alcopops were not positively associated with increased risk of any alcohol-related harms (Table 4).

With our results showing cheaper alcohol products linked most strongly to adverse drinking outcomes and other work identifying underage alcohol consumption being sensitive to price [42], governments should establish a minimum price for alcohol (per unit). Drinking bottles and cans of beer was also linked to violence, regretted sex and public drinking while alcopops and wine appeared protective against alcohol-related violence and public drinking respectively (Table 4). Although it is possible to speculate that such effects may relate to the image of each product (e.g. beer may be considered a drink for tougher youths than alcopops) or the location in which such drinks are consumed (e.g. wine may be more likely to be consumed in moderating environments such as at home with parents) understanding such factors requires further investigation [43].

As with any questionnaire based cross-sectional study this survey has a number of limitations. Both drinking behaviours and negative outcomes were self-reported and relied on the honesty and recollection of respondents [44]. Whilst guaranteed anonymity can encourage the former, our results establish that recollection of behaviours relating to alcohol consumption may be incomplete because of forgetting things after drinking, especially amongst those who binge (Table 4). Calculations of units of alcohol consumed per week could only be broad approximations as a wide variety of products are available and our calculations are based on individuals classifying their drinking according to only seven general product descriptions. In particular, estimates for alcopops assume a volume of 275 ml for each bottle consumed but 700 ml bottles are now stocked in a number of outlets. Moreover, while the survey specifically examined alcohol-related outcomes (e.g. violence when drunk), it did not provide information on the amount individuals had consumed precisely when such outcomes occurred but only measured their current typical drinking patterns. Consequently, we cannot rule out that some adverse drinking behaviours may have developed as a coping mechanism after, for instance, being a victim of alcohol-related violence or regretted sex [45,46]. Sampling did not include individuals who were excluded from or had otherwise left school-based education, and deprivation was assigned on an ecological basis rather than through individual circumstance. Analyses did not account for potential effects relating to variance at school level but did include deprivation as a measure of community level effects. Adverse effects of alcohol were limited to four measures and did not include correlates with prevalence of injury (e.g. hospital attendance) or other potential consequences (e.g. effects on education, relationship problems) [15,47]. However, chosen outcomes did include adverse measures previously associated with males (violence) [29], an adverse sexual outcome linked to alcohol (regretted sex) [30,31], a measure of potential damage to mental health and development (forgetting things after drinking) [32] and a proxy for involvement in public nuisance (drinking in public places). Finally, no quantitative measures of compliance were collected from schools and although response rates were high for most questions (>85%), for those drinking at least weekly responses only allowed calculation of units consumed per week in 81.2% of cases. Thus, some selection bias effects could not be ruled out and consequently we have not extrapolated results to population levels.

## Conclusion

Our results support those of others that suggest even low levels of consumption can not be considered safe for children [11]. While studies suggest that levels of youth alcohol consumption may be high in England, and especially in the North West region [48], the reality in many countries is that by the ages of 15 and 16 a higher proportion of children drink alcohol than abstain [15,16]. Any efforts to move more children towards or into abstinence through parental rules and controls may be effective for some individuals [26,27], but may also result in alcohol consumption moving out of the family environment into parks, streets or other public spaces. Our results suggest that such a move, even if overall consumption did not increase, could exacerbate negative outcomes from alcohol consumption amongst teenagers. More studies and meta-analyses are needed to refine public information on alcohol consumption by children. Our results, nevertheless, do suggest that those parents who allow children aged 15-16 years to drink may limit harms by restricting consumption to lower frequencies (e.g. no more than once a week) and under no circumstances permitting binge drinking. However, parental efforts should be matched by genuine legislative and enforcement activity to reduce independent access to alcohol by children, and examination of costs per unit and bottle sizes to discourage large bottle purchases. While these measures are unlikely to eradicate the negative effects of alcohol on children, they may reduce them substantially while allowing children to prepare themselves for life in an adult environment dominated by this drug.

## Competing interests

The authors declare that they have no competing interests.

## Authors' contributions

MAB contributed to study design, analysed the data, and wrote the manuscript. PAPH assisted MAB in developing concepts and writing the manuscript. KH and SH contributed to study design and co-ordination, and commented on the manuscript. MM coordinated data collection from licensed premises. PAC, MM, and LJ assisted in the production of the manuscript. KH contributed to data analysis and commented on the manuscript. LS conducted the survey. All authors read and approved the final version.

## Pre-publication history

The pre-publication history for this paper can be accessed here:


